# The functional sphincter of Oddi disorder

**Published:** 2008-04-15

**Authors:** Pop Corina, Purcăreanu Adina, Purcărea Monica, Andronescu Dan

**Affiliations:** * Department Of Gastroenterology And Internal Medicine University Hospital Bucharest

**Keywords:** sphincter of Oddi, functional disorder, sphincter of Oddi dysfunction, manometry, Oddi disorder

## Abstract

The sphincter of Oddi disorder (SOD) has been a controversial subject for many years, about which a lot has been written. However, new findings mainly using Endoscopic Retrograde Cholangiopancreatography (ERCP) and sphincter of Oddi manometry (SOM) demonstrate the fact of this diagnostic. SOD is just a part of a larger pathology, the functional gastrointestinal disorders, which have been reconsidered as an important part of gastrointestinal diseases. For a better understanding, the American Gastroenterology Association Institute created a new classification of The Functional Gastrointestinal Disorders in 2006, Rome III Classification, in which the SOD is grouped in the functional biliary disorders (category E).

The term SOD is used to define manometric abnormalities in patients who have signs and symptoms consistent with a biliary or pancreatic ductal origin. Based on the pathogenic mechanism and manometry findings, the SOD is separated into two groups: a group characterized by a stenotic pattern (anatomical abnormality) and a second group with a dyskinetic pattern (functional abnormality).

The purpose of this article is to construct a short presentation of the main aspects regarding functional SOD (E2 and E3 after Rome III Classification).

## Introduction

Functional biliary disorders are common gastrointestinal diseases seen in general practice and by the gastroenterologist. They are clinically characterized by biliary-type symptoms and include gallbladder dysmotility and the sphincter of Oddi dysfunction.

Sphincter of Oddi dysfunction is a structural or functional abnormality of the sphincter’s motility that interferes with bile or pancreatic duct drainage [**1**]. Therefore, the SOD is associated with abdominal pain, elevated liver/pancreatic enzymes, common bile duct dilatation or episodes of pancreatitis.

Sphincter of Oddi dysfunction refers to two distinct pathological conditions based upon their distinctly pathogenic mechanism, sphincter of Oddi stenosis, anatomical abnormality (which is permanent raised sphincter base pressure) and sphincter of Oddi dyskinesia, functional abnormality (which is a paradoxical response to cholecystokinin injection, high contraction frequency, high percentage of retrograde contractions or short periods of raised base sphincter pressure) [**4**].

In the case of sphincter stenosis, the sphincter is narrowed by inflammation and fibrosis secondary to different affections such as intraoperative trauma, pancreatitis, passage of biliary gallstone, adenomyosis and infections (especially in AIDS patients, infections with Cytomegalovirus, Cryptosporidium or Strongyloides). Sphincter stenosis is associated with abnormal SO motility and permanent elevated base pressure, that does not respond to smooth muscle relaxants.

Sphincter of Oddi dyskinesia is characterized by intermittent obstruction of the sphincter and includes all motility abnormalities of the sphincter. The sphincter dyskinesia is less well reproduced upon repeated measurement than sphincter stenosis and can result in a paradoxical response after cholecystokinin administration.

The main group of symptoms is the same for both entities, including recurrent biliary or abdominal pain [**5**].

Due to the important impact of the functional gastrointestinal disorders on general population, the American Gastroenterology Association created in 2006 the last classification of functional gastrointestinal disorders called Rome III Classification (which replaced the previous Rome II Classification from 1999). In Rome III Classification, functional gastrointestinal disorders are grouped into six major domains for adults (category A-F) and two pediatric domains (category G-H). The domains for adults are: oesophageal (category A), gastroduodenal (category B), bowel (category C), functional abdominal pain syndrome (category D), biliary (category E) and anorectal (category F). The biliary functional disorders (category E) contain: functional gallbladder disorder (E1), functional biliary sphincter of Oddi disorder (E2) and functional pancreatic sphincter of Oddi disorder (E3) [**6**]. 

## Anatomy and physiology

The sphincter of Oddi represents a segment of smooth muscle situated at the junction of the common bile duct with the main pancreatic duct.

The first who described this entity was British anatomist Francis Glisson (1595–1677) who carried out an important work on the anatomy of the gastrointestinal tract, especially of the liver [**7**]. However, Ruggero Oddi, Italian anatomist, whilst still a student, was the one who described it as a structure with distinct physiological properties in 1887, which is why was later, it known as the eponymous “Sphincter of Oddi” [**8**].

Edward A. Boyden studied this sphincter extensively and established that the embryologic development of the sphincter of Oddi is distinct from the muscular fibers of the duodenum, having its origins in the local mesenchyme.[**9**] Anatomically, Boyden described three groups of muscle fibers: the sphincter of choledochus (with a superior and an inferior part), the sphincter of the pancreatic duct and the sphincter of the papilla (ampulla) [**10**]. Boyden also individualized the preampullar part of the sphincter as the principal muscle involved in spilling the bile into the duodenum (part called the Boyden’s sphincter) [**11**].

In 1971, Baraya distinguished three segments of the sphincter of choledochus: superior (outside the duodenum), medium (with a length of 15mm) and inferior. The junction between the medium and the inferior segments continue with that of the ampulla. In the same way, the sphincter of the pancreatic duct is made up of three segments and the inferior segment is linked with the muscular fibers of duodenum [**12**]. This entire structure is not constant, being different according to the type of union between the choledochus and the pancreatic duct.

The sphincter of Oddi is contained almost entirely within the duodenal wall, following an oblique course on a length of about 6-10mm [**13**].

The function of sphincter of Oddi is: to regulate bile and pancreatic exocrine juice flow into the duodenum, to maintain resistance to the bile flow and in this way to allow gallbladder filling during fasting, to maintain sterile intraductal environment by preventing duodenal biliary reflux.

For this purpose the sphincter of Oddi has a base pressure as well as a phasic contractile activity that have been demonstrated by endoscopic manometry [**14**].

The base pressure of the sphincter of Oddi maintains a difference of approximately 15mmHg between the duodenum and the biliary or pancreatic duct, in this way also creating a sterile intraductal environment. The flow of bile and pancreatic juice into the duodenum is regulated by prominent phasic contractions. The phasic contractile activity of the sphincter of Oddi has a peak of 100-150/mmHg, has a frequency of four - five contractions per minute on average and the duration is roughly four - six seconds [**15**].

The majority of contractions are oriented anterogradel and facilitates the flow of bile and pancreatic juice into the duodenum. Some of the phasic contractions are spontaneous and retrograde being partially involved in the pathogenesis of the sphincter of Oddi disorder.

In SOD the base sphincter pressure is more than 40mmHg, the phasic contractions are more than 350mmHg with a frequency of more than seven contractions per minute. More than 50% of the phasic contractions are retrograde propagation sequences [**16**, **17**].

The phasic contractile activity of the sphincter of Oddi is correlated with the migrating motor complex of duodenum during fasting and the frequency of phasic contractions increases just before phase 3 of the duodenal activity [**18**]. This allows the coordinated release of bile into the duodenum, this being an important mechanism because the bile flow during fasting can prevent bile crystal formation [**19**]. After feeding the amplitude of contractions decreases and the sphincter tonus is reduced [**20**].

The sphincter of Oddi activity has both a hormonal and a neural influence. Almost three quarters of the gallbladder volume emptying takes place during the meal, under the influence of cholecystokinin and the rest during fasting, with the influence of the migratory motor complex [**21**]. Therefore, cholecystokinin is the main stimulus for gallbladder contraction and sphincter of Oddi relaxation [**22**]. After cholecystectomy, it has been demonstrated manometrically that the normal inhibitory effect of pharmacological doses of cholecystokinin on sphincter of Oddi is suppressed, mechanism involved in sphincter of Oddi postcholecystectomy dysfunction [**23**]. Beside cholecystokinin, another important hormonal factor is secretin, this being involved in stimulating the pancreatic secretion and relaxing the sphincter of Oddi [**34**].

The baseline sphincter of Oddi phasic activity and duodenal motility regulation involves the generation of NO from L-arginine, being regulated by cholinergic stimulatory and NO-mediated inhibitory neural pathways [**25**].

In the normal sphincter of Oddi and duodenum there are abundant nitric oxide and also vasoactive intestinal peptide positive innervations. It has been demonstrated that the lower proportion of nitric oxide peptide positive or vasoactive intestinal positive nerve cells of the disease group may cause an inadequacy of the sphincter of Oddi relaxation [**26**].

The sphincter of Oddi can be also relaxed by atropine, glucagon, nitroglycerin and calcium channel antagonist and stimulated by narcotic agents [**27**].

The afferent pathways for the motor reflex response during digestion are represented by the fibers of vagus, while the efferent pathways are provided by the splanchnic fibers. The sympathetic fibers from the splanchnics are mostly inhibitory and the parasympathetic fibers from the vagus have a stimulating effect on the motor activity of gastrointestinal organs [**28**].

Even though there is a close relation between motor activity of the gallbladder and the sphincter of Oddi, the innervations seem not to be essential for its motility, as demonstrated by the preserved function after liver transplant [**29**].

## Epidemiology

The sphincter of Oddi disorder affects patients of any age and gender. The studies on functional gastrointestinal disorders showed that females are diagnosed with the sphincter of Oddi dysfunction more frequently than males and that middle – age females are predisposed [**30**].

The sphincter of Oddi dysfunction explains part of the symptoms generally called biliary dyskinesia. Out of 34 patients with biliary dyskinesia who had undergone clinical and fibrogastroduodenoscopic examinations, fractional duodenal intubations with examination of the bile, ultrasonography and dynamic nuclear scintigraphy of the hepatobiliary system, 28% had the sphincter of Oddi hypertension and 44% had sphincter of Oddi hypotonia [**30**].

The sphincter of Oddi dysfunction was commonly described after cholecystectomy (hence the name of postcolecystectomy syndrome) [**32**] and about 10 - 20% of the patients with cholecystectomy continue to exhibit abdominal pain [**33**,**34**]; the symptomatology may be similar with the preoperative one or may be different [**34**]. Also, the sphincter of Oddi dysfunction may occur in patients with gallbladder in situ and can be one of the reasons that determine cholecystectomy [**35**].

Another category of patients diagnosed with the sphincter of Oddi dysfunction were those with recurrent pancreatitis and pancreatic-type pain determined by the dysfunction in the pancreatic duct portion [**36**].

Out of 360 patients with functional gastrointestinal disorders, the sphincter of Oddi dysfunction was diagnosed in 61%. The most frequent was abnormal basal pressure for both biliary and pancreatic sphincters found in 31% of the cases; the next affected was pancreatic sphincter alone in 19% and the abnormal biliary basal sphincter pressure alone was found in 11 % [**37**].

## Clinical presentation and diagnostic methods

All the functional GB and SO disorder (biliary SO or pancreatic SO) have almost the same clinical presentation. In all cases, abdominal pain is the most common symptom.

**Table 1:** Rome III Classification Functional Gastrointestinal Disorders

A	Functional Esophageal Disorders
B	Functional Gastroduodenal Disorders
C	Functional Bowel Disorders
D	Functional Abdominal Pain Syndrome
E	Functional Gallbladder and Sphincter Oddi Disorders
F	Functional Anorectal Disorders

The Rome III Classification summarizes the abdominal pain characteristics necessary to diagnose the functional GB and SO disorder: site (located in the epigastrium and/or right upper quadrant), severity (the intensity of pain must be high: daily activities are disturbed and the patient sees the doctor for it), duration (episodes of pain last at least 30 minutes, recurring at different intervals). Other functional diseases (the pain is not relieved by bowel movement, postural change or antacids) or structural diseases (cholelithiasis, gastroesophageal reflux disease, pancreatitis) with similar range of symptoms should be excluded.

**Table 2:** Rome III Classification Functional Gallbladder and Sphincter Oddi Disorders

E1	Functional Gallbladder Disorders
	- all of the following
	• criteria for functional GB and SO disorders
	• GB is present
	• normal liver enzymes, conjugated bilirubin, and amylase/lipase
E2	Functional Biliary Sphincter Oddi Disorders
	- both of the following
	• criteria for functional GB and SO disorders
	• normal amylase/lipase
	- supportive criterion
	• elevated serum trasaminase, alkaline phosphatase or conjugated bilirubin temporally related to at least two pain episodes
E3	Functional Pancreatic Sphincter Oddi Disorders
	- both of the following
	• criteria for functional GB and SO disorders
	• elevated amylase/lipase

The diagnostic criteria for the abdominal pain in functional GB and SO disorder, that are all necessary, are completed by one or more of the supportive criteria, that include nausea and vomiting, pain that radiates to the back (pancreatic SOD) and right infrascapular region (biliary SOD) or appears during sleep [**38**].

Rome III Classification can not be used on its own for positive diagnosis; clinical evaluation of the patient should be completed with serum biochemistry and imagistic explorations.

**Serum biochemistry**

Abnormal serum liver and pancreatic enzymes can be found in patients with SOD, especially during episodes of abdominal pain. Even the sensibility and specificity of the serum enzyme is low, the elevated serum aminotransferases (ALT and AST) in patients with biliary SOD and amylase or/and lipase in patients with pancreatic SOD can be used in choosing the appropriate therapeutic method. Abnormal liver tests may indicate the advantageous response to endoscopic sphincterotomy, as demonstrated by Lin et al on Geenen-Hogan class II postcholecystectomy patients with abnormal liver function tests (90% were pain-free after sphincterotomy) [**39**].

**Pain provocative test**

The Nardi test (named after George Nardi, who first described the procedure in 1966), also known as the morphine-prostigmine provocation test (an intramuscular injection of 10mg of morphine and 2mg of prostigmine), was used to stimulate the spasm of SO and the production of exocrine pancreatic secretion. Elevation of liver or/and pancreatic enzyme or reproduction of the pain represent a positive test, confirm the SOD diagnosis and also the clinical advantage after sphincterotomy. Because of the low sensitivity and specificity, this test is no longer used [**40**]. 

**Ultrasonography (US)**

The common bile duct diameter can be measured using transabdominal US, dilatation above 6mm indicates the increased resistance to bile flow and is likely to be a feature of SOD [**41**]. This analysis is not enough because dilatation of the common bile duct diameter can also be found in elderly persons (with the upper normal limit at 8.5mm) [**42**] and in asymptomatic cholecystectomized patients [**44**].

Provocation tests have been developed to observe the motility of SO and to increase the specificity of US examination. After a fatty meal or after cholecystokinin administration, bile flow increases, but common bile duct diameter remains constant in normal SO function. A diameter increase of more than 2mm is considered pathological and reflects the raised resistance to bile flow [**44**].

The pancreatic duct diameter can also be determined using transabdominal US; normal average values are 3mm in the area of the head-neck, 2.1mm in the body proximal and 1.6mm in the body distal to the neck [**45**]. In normal patients, the pancreatic duct diameter increases and then returns to normal in less than 15 minutes. An increase in the pancreatic duct diameter of more than 1.5mm for more than 30 minutes after a fatty meal or after secretin infusion is considered pathological [**45**].

Endoscopic ultrasonography and computer tomography were used to increase the sensibility of the ductal diameter measurement with good results [**47**, **48**].

**Magnetic resonance cholangiopancreatography (MRCP)**

MRCP is important in determining the presence of structural obstructive causes (stones, tumors) and is the least invasive method to obtain a cholangiogram or a pancreatogram [**49**]. MRCP has been used to evaluate the dynamic anatomy of pancreatic duct after secretin infusion in patients with recurrent episodes of pancreatitis [**50**].

**Hepatobiliary scintigraphy (HS)**

With HS using technetium-99m, patients with SOD present delayed biliary drainage into the duodenum. In order to increase accuracy, HS can be combined with provocative tests. There were studies that recommended it for diagnosis for its good correlation with manometry [**51**]. Results of other studies indicated that the method had a relatively low sensitivity and sensibility and correlated poorly with manometry, not being recommended for general clinical use [**52**]. Therefore, new studies are necessary in order to reach a conclusion.

**Endoscopic retrograde cholangiopancreatography (ERCP)**

ERCP alone is no longer recommended for evaluating the SOD given the comparable sensitivity

with noninvasive methods like US or MRCP [**53**]. 

**Sphincter of Oddi manometry (SOM)**

SOM remains the most important diagnostic method for SOD. SOM is done during ERCP with a water-perfused pressure-sensitive catheter that is passed into the sphincter through an endoscope that has been placed into the duodenum. The catheter is slowly withdrawn from the bile duct into the duodenum through the SO, whilst recording the basal sphincter pressure and the phasic contractions in different parts of the bile duct (proximal, medium and distal) and in the duodenum. The abnormally high basal sphincter pressure, more than 40mmHg, is the only criteria accepted for diagnosing the SOD by most authors. Other abnormalities that can be found in patients with SOD are an increase in retrograde SO phasic waves, high frequency of phasic contractions, paradoxical response to cholecystokinin or other smooth muscle relaxants [**54**].

If pressure recording suggests SOD, proper therapy involves an endoscopic cutting of the sphincter (sphincterotomy). In a controlled study of patients with suspected type II biliary SOD, the response rate after sphincterotomy was 91% when basal pressure was greater than 40mmHg and only 42% with a normal SO pressure [**55**]. This good response after sphincterotomy for patients with increased basal sphincter pressure indicates a suggestion of therapy. There were studies that recommended other criteria, like elevated liver enzymes and biliary dilatation, obtained through noninvasive methods, as better predicting the response to sphincter ablation [**56**]. Recently, injection of botulinum toxin into the SO, which inhibits the motility of the sphincter for almost 3 months, has been used to select patients who will respond to sphincterotomy [**57**]. 

Measuring only the basal pressures from either the biliary or pancreatic ducts is not recommended because the separate dysfunction may be found in almost 25% of patients [**58**]. In the evaluation of patients with recurrent pancreatitis, normal biliary SO was found in the presence of abnormal pancreatic SO [**59**]. Another study that evaluated 68 patients found the following results: of all patients diagnosed with SOD, 29% had chronic pancreatitis and of those diagnosed with chronic pancreatitis, 87% had SOD [**60**].

Even if SOM is the main method for SOD diagnosis, it has some limitations. Besides the fact that it is not largely available, SOM increases the risk of ERCP-associated pancreatitis. The incidence of pancreatitis was 17% in a study of 100 consecutive patients who underwent sphincter of Oddi manometry, that percentage being greater compared with the one using ERCP only [**61**].

Rome (I, II, III) Classification is used as the main diagnosis criteria for the abdominal pain in SOD, a subjective symptom. Also, the characteristic of abdominal pain in SOD is similar with symptoms of other structural or functional gastrointestinal disorders. So, another classification using objective clinical features is necessary to complete the evaluation. 

**Table 3:** Rome III Classification Diagnostic Criteria for Functional GB and SO Disorders

Pain located in the epigastrum and/ or upper quadrant and all 8 points	1. Episodes lasting 30 minutes or longer
	2. Recurrent symptoms occurring at different intervals (not daily)
	3. The pain builds up to a steady level
	4. The pain is moderate to severe enough to interrupt the pacient’s daily activities or lead to an emergency department visit
	5. The pain is not relieved by bowel movements
	6. The pain is not relieved by postural change
	7. The pain is not relieved by antacids
	8. Exclusion of other structural disease that would explain the symptoms
Supportive criteria one or more of 3	1. Pain is associated with nausea and vomiting
	2. Pain radiates to the back and/or right infrasubscapular region
	3. Pain awakens from sleep in the middle of the night

The Classical Milwaukee Biliary Group Classification (Hogan-Geenen classification) for biliary SOD [**62**] and The Modified Milwaukee Biliary Group Classification for pancreatic SOD [**63**] recognize three biliary, and three pancreatic subtypes respectively.

All subtypes exhibit abdominal pain (biliary or pancreatic pain) as first objective clinical criteria. Also, abnormal liver/pancreatic enzymes are elevated (twice as high as normal on at least two occasions). Another objective criterion is delayed drainage of ERCP contrast for more than 45 minutes and dilated common bile duct more than 12mm for biliary SOD.

ERCP is an invasive diagnosis method that could have important complications like ductal or pancreatic tissue damage. That is why, in order to avoid early ERCP, the Rome III consensus statement revised The Classical Milwaukee Biliary Group Classification (Hogan-Geenen classification). The revized Classification uses US, noninvasive method, to evaluate the common bile duct diameter. 

**Table 4:** Biliary SOD

	Milwaukee Biliary Group Classification	Revized Milwaukee Biliary Group Classification The Rome III consensus statement
type III	• biliary-type pain	• recurrent biliary-type pain
type II	• biliary-type pain and *one or two* of the following	• biliary-type pain and *one* of the following
	• abnormal liver function tests (>2 times normal)	• abnormal – aminotransferases - bilirubin or - alkaline phosphatase (>2 times normal on at least 2 ocassions)
	• dilated common bile duct (>12mm)	• dilated bile duct (> 8mm)
	• delayed dranaige of ERCP contrast ( >45 minutes)	
type I	• biliary-type pain and *all* of the following	• biliary-type pain and *all* of the following
	• abnormal liver function tests (>2 times normal)	• abnormal – aminotransferases - bilirubin or - alkaline phosphatase (>2 times normal on at least 2 ocassions)
	• dilated common bile duct (>12mm)	• dilated bile duct (> 8mm)
	delayed dranaige of ERCP contrast ( >45 minutes)	

Also, the Rome III consensus statement has developed a similar classification system for pancreatic SOD. This new classification, used to investigate patients with abdominal pancreatic pain or recurrent pancreatitis, evaluates the serum pancreatic enzymes, the pancreatic duct diameter and the time of contrast drainage after ERCP.

The subtypes of The Classical Milwaukee Biliary Group Classification are correlated with the positive diagnosis of SOD through SOM: from 80-90% for type I biliary SOD to only 7-30% for type III biliary SOD and from 90% for type I pancreatic SOD to only 35% for type III pancreatic SOD [**64**, **65**].

**Table 5:** Pancreatic SOD

	Modified Classification of pancreatic type SOD	Revized Classification of pancreatic type SOD The Rome III consensus statement
type III	• pancreatic-type pain	• pancreatic-type pain
type II	• pancreatic -type pain and *one or two* of the following	• pancreatic -type pain and *one or two* of the following
	• amylase/ lipase (>1.5 - 2 times normal)	• amylase/ lipase (>1.5 times normal)
	• pancreatic duct > 6mm in head or >5mm in body	• pancreatic duct > 6mm in head or >5mm in body by ERCP
		• delayed drainage of contrast after ERCP (>9 minutes)
type I	• pancreatic -type pain and *all* of the following	• pancreatic -type pain and *all* of the following
	• amylase/ lipase (>1.5 - 2 times normal)	• amylase/ lipase (>1.5 times normal)
	• pancreatic duct > 6mm in head or >5mm in body	• pancreatic duct > 6mm in head or >5mm in body by ERCP
		• delayed drainage of contrast after ERCP (>9 minutes)

## Treatment

The purpose of therapy in SOD is to reduce sphincter resistance to bile and/or pancreatic juice flow. For this, medical therapy and also endoscopic and surgical procedures have been developed.

As medical therapy, spasmolytic agents (calcium channel blockers and nitrates) were studied for the management of SOD in relation with SO smooth muscle structure.

Nitrates (sublingual nitroglycerine) were used to relax SO and, in this way, to reduce basal sphincter pressure [**66**].

Calcium channel blockers (sublingual nifedipine) have been proved to reduce the intensity of abdominal pain to approximately 75% of patients with increased basal sphincter pressure at SOM [**67**].

The use of medical therapy is considered in all type III SOD and in less severe type II SOD because it may have frequent side-effects. It may not be beneficial in SO stenosis and also has an incomplete response in patients with primary motor abnormality of the SO.

The most effective therapy in SOD is total division of SO [**68**]. Because pain is produced by sphincter spasm, division of the sphincter should stop the episodes of pain and allow a good drainage of pancreatic bile and pancreatic juice into the duodenum. Sphincterotomy can be realized by transabdominal approach or by endoscopy.

The surgical transduodenal approach is the traditional curative method for SOD. The most common surgical intervention used was transduodenal sphincteroplasty with transampullary septectomy [**69**]. For the surgical therapy, rates of response were correlated with the basal sphincter pressure: patients with an elevated basal sphincter pressure determined by intraoperative SOM were more likely to improve after surgical ablation than those with a normal basal pressure [**70**]. There were studies that suggested that the use of surgical therapy for biliary SOD had a better outcome than for pancreatic SOD, however, these conclusions were not sustained by other studies [**71**].

The advantages of surgical sphincterotomy compared with endoscopic sphincterotomy are a better efficiency due to a good access of the transampullar septum and a lower rate of recurrent stenosis [**72**]. Surgical approach also has its disadvantages: it is more invasive, associated with an increased rate of morbidity (like postoperative pancreatitis), mortality, and cost of care. Also, patient tolerance and cosmetic results are better for the endoscopic approach [**73**].

Nowadays, surgery has been replaced by endoscopical division of the SO. Surgical therapy is, at present, a solution for cases of restenosis following endoscopical treatment only.

Multiple studies have evaluated endoscopic sphincterotomy for SOD. The results recommended the endoscopical management as the standard therapy in SOD for the initial invasive treatment.

Patients with an elevated basal pressure (greater than 40mmHg) seem to have the greatest benefit extended out to 4 years [**74**].

Another study indicates a tendency for patients with bile ducts diameter greater than 12mm to have a sustained clinical improvement after endoscopic sphincterotomy [**75**].

Results from a 5-year prospective clinical trial on one-hundred and eight patients with recurrent biliary-type I and type II pain after cholecystectomy, indicate that the Geenen-Hogan classification helps predict the clinical outcome after endoscopic sphincterotomy [**76**]. Another study recognizes that endoscopic sphincterotomy provides symptomatic relief for the majority of patients but also that improved criteria for predicting the outcome are required [**77**].

Drainage of the biliary or pancreatic duct through a stent has also been used with varied results in different studies. Some results indicate increased ductal and parenchymal injury after stent utilisation and others recommend the use of stents in SOD [**78**, **79**]. The use of a small diameter stent for a short period of time has reduced the incidence of post-ERCP pancreatitis [**80**].

Balloon dilation of SO was developed in order to obtain a less invasive approach of SOD and to preserve the SO function. Because of the high rate of complications, the use of this method was acceptable only in patients who have coagulopathy, who are at risk for infection, and possibly those who are older [**81**]. 

## Conclusion

The functional sphincter of Oddi disorder represents a combination of motility abnormalities that are the source of significant clinical symptoms. Manometry is a good instrument of diagnosis, but noninvasive methods of diagnosis for SOD should be developed in the future. Also, an ideal therapeutic option has not been found yet. This way, even having answers to a lot of questions regarding SOD, it remains still a chapter open for study.

## Abbreviations used

ERCP - endoscopic retrograde cholangiopancreatography; ES - endoscopic sphincterotomy; GB – gallbladder; MRCP - Magnetic resonance cholangiopancreatography ; SO - sphincter of Oddi; SOD - sphincter of Oddi disorder; SOM - sphincter of Oddi manometry; US – ultrasonography
